# Resistance-based exercise restores muscle health in dialysis patients

**DOI:** 10.1007/s10974-026-09727-0

**Published:** 2026-03-14

**Authors:** Piush Srivastava, Sunil Kumar Singh, Rakesh Sathish Nair, Saket Jha, Navin Viswakarma, Deepti Srivastava, Greg Hachaj, Stephen T. Bartlett, Ilaria Santi, Aslam Ejaz, Robert W. Motl, Enrico Benedetti, Sandeep Kumar, Ajay Rana

**Affiliations:** 1 Department of Surgery, College of Medicine, University of Illinois Chicago, 840 S. Wood Street, Chicago, IL 60612 USA; 2 Health Sciences System Cancer Center, University of Illinois Hospital, University of Illinois Chicago, Chicago, IL 60612 USA; 3 Department of Kinesiology and Nutrition, College of Applied Health Sciences, University of Illinois Chicago, Chicago, Chicago, Illinois, IL 60612 USA; 4 Research Unit, Jesse Brown VA Medical Center, Chicago, IL 60612 USA

**Keywords:** Dialysis, Frailty, Skeletal muscle, Personalized exercise, Muscle regeneration, Muscle function

## Abstract

Chronic kidney disease (CKD) and end-stage renal disease (ESRD) are serious health concerns worldwide that require regular dialysis before a kidney transplant for a better quality of life. Due to aging and frailty, dialysis patients are often limited in physical activity, which can lead to multiple health complications and sometimes result in patient death. Therefore, maintaining muscle health is crucial for individuals undergoing dialysis. We recently reported that personalized resistance-based exercise can improve frailty in dialysis patients; however, the molecular mechanisms underlying its effects on muscle health remain unknown. RNA-Seq analysis revealed that exercise upregulates genes associated with muscle stemness, regeneration, and differentiation. Furthermore, our study demonstrated that exercise upregulates mitochondrial proteins involved in the oxidation of organic compounds and mitochondrial function in muscle. Moreover, we observed that exercise increased energy production via glucose uptake, glycogenesis, and fatty acid metabolism. These results suggest that personalized resistance-based muscle therapy in dialysis patients restores the expression of genes and proteins associated with muscle stemness, regeneration, differentiation, and energy generation.

**Supplementary Information:**

The online version contains supplementary material available at 10.1007/s10974-026-09727-0.

## Introduction

The patients with end-stage kidney disease (ESRD) require either dialysis or a kidney transplant to survive (Lange-Maia et al. 2022). Kidney replacement therapy, including kidney transplants, is estimated to be approximately 4.9-7 million worldwide (Lv and Zhang 2019). For patients with ESRD, dialysis or kidney transplants are necessary to filter blood and remove toxins and excess fluids from the body. Dialysis is considered an immediate solution and a life-sustaining treatment, whereas a kidney transplant is a long-term solution for patients (Grinyo 2013). However, a kidney transplant is not an easy option, because it’s a time-consuming and costly procedure, and therefore most of the kidney patients rely on dialysis.

Although dialysis is the convenient option for patients with ESRD, it is associated with some side effects as well (Hassan et al. 2021; Herreros-Carretero et al. 2024). For example, patients with dialysis have a high prevalence of musculoskeletal disorders (Hassan et al. 2021; Herreros-Carretero et al. 2024). Severe musculoskeletal disorders associated with loss of lean mass can lead to disability in movement (Salaffi et al. 2020). If untreated, musculoskeletal frailty can lead to severe conditions, including death, in dialysis patients (McAdams-DeMarco et al. 2015). Muscle frailty is the loss of muscle mass and strength due to aging and certain health conditions (Prell et al. 2024). To address frailty, two approaches are commonly employed: first, the use of protein supplements, and second, exercise to strengthen muscles, also known as muscle-strengthening exercise (MSE) (Liao et al. 2019). Recently, we conducted a randomized clinical trial in dialysis patients to investigate the reversibility of frailty through customized muscle-strengthening exercises (Bartlett et al. 2025). Notably, one year of muscle rehabilitation therapy in dialysis patients indicated noticeable improvements in their physical capacity (Bartlett et al. 2025). These exercise outcomes suggested that the customized muscle-specific exercise could be a practical approach to improving frailty in dialysis patients.

We noted corresponding improvements in morphological changes in the muscles of dialysis patients after the muscle-specific exercise (Bartlett et al. 2025). However, an in-depth assessment of molecular changes in muscles associated with customized muscle-strengthening exercise was not reported. Therefore, we sought to comprehend the status of various gene signatures related to muscle health. In the present study, RNA-Seq, immunohistochemistry (IHC), and immunofluorescence (IF) analysis revealed that exercise restores the expression of genes associated with muscle regeneration, differentiation, and energy synthesis. Furthermore, our study demonstrated that customized muscle-strengthening exercises improve mitochondrial function in muscle and increase energy production through the oxidation of organic compounds, primarily via glucose uptake, glycogenesis, and fatty acid metabolism.

## Materials and methods

### Muscle samples from the dialysis patients

Muscle biopsies were obtained from dialysis patients under our approved IRB protocol. Dialysis patients consented to an optional biopsy of the forearm flexor muscle at baseline and 12 months after resistance-based muscle therapy, as described in detail in our recent publication (Bartlett et al. 2025). A brief detail of the study is given below:

#### Study design

This study was designed to evaluate the effects of a specific muscle rehabilitation program comprising 12 months of low-intensity, personalized strength intervention. The intervention was conducted on debilitated pre-transplant patients with kidney failure, who were on dialysis, and were being evaluated for organ transplantation. The study was approved by the Institutional Review Board.

#### The primary and the secondary outcomes of the study

**(A)** The primary outcome of the study was the Short Physical Performance Battery (SPPB). The SPPB test includes a summary of three separate measures: walking speed, balance, and timed sit-to-stand. Gait speed was determined by asking participants to walk 2.44 m as quickly and safely as possible. Subjects who were unable to walk were assigned a speed of 0.01 m/s. To perform the balance test, subjects must stand in 3 positions for 10 s each (tandem, semi-tandem, and side-by-side). For the Timed Seated Chair Stand, participants were asked, when possible, to stand up and sit down from a chair without using their arms for a total of 5 times. The time the subject took to complete 5 sit-to-stands was recorded in seconds. If participants were unable to perform this test, they were assigned a time of 32 s, corresponding to the 99th percentile among patients who could perform it. The total scoring for the SPPB test was based on a scale of 0–12, with lower numbers representing worse physical performance and greater frailty. **(B)** Secondary outcomes included handgrip strength, mental and physical assessments using validated surveys, and an optional muscle biopsy. The hand grip strength was measured in kilograms using a hand dynamometer (Jamar Hydraulic Hand Dynamometer, model 5030J1). Participants were asked to perform the test with their dominant hand, and muscle contractions were elicited in a seated position with the dominant arm at 90 degrees. Grip strength was recorded as the average of 3 attempts. The 36-item Short Form Survey includes 8 domains: physical functioning, bodily pain, role limitations due to physical health problems, role limitations due to personal or emotional problems, emotional well-being, social functioning, energy/fatigue, and general health perceptions. Scoring is on a scale of 1-100, with higher scores indicating a more favorable overall health state. The Patient-Reported Outcomes Measurement Information System (PROMIS)-29 global health survey includes items on depression, anxiety, physical function, pain interference, fatigue, sleep disturbance, and ability to participate in social roles and activities, along with a single item on pain intensity.

#### Sample size

For this specific manuscript, only patients who consented to the optional muscle biopsy were included.

#### Inclusion and exclusion criteria

Subjects were included in the study if they were patients on hemo- or peritoneal dialysis, or had a glomerular filtration rate < 20 mL/min, and were undergoing evaluation for kidney transplantation. Participants were excluded if they were aged < 18 years, had cardiac or pulmonary disease contraindicating exercise training, or were unable to cooperate with protocol procedures.

Inclusion Criteria:

- Clinically debilitated patients, currently on dialysis, or who have a glomerular filtration rate < 20 mL/min.
- Adequate cognitive ability to complete the questionnaires, give consent for the study, and follow the instructions.
- 18 years of age and older.

Exclusion Criteria:

- cardiac/pulmonary disease that contraindicates the physical training.
- unable to comply with the training program.
- vulnerable population, including pregnant women, minors, decisionally impaired, and prisoners.

#### Patient’s evaluation

All patients were evaluated using the mentioned techniques for the primary and secondary outcomes. All participants have been tested at baseline, 6 months, and 12 months using the exact same battery of tests. If this refers to the techniques used to analyze muscle samples, only those who consented to the optional biopsy were evaluated. We didn’t ask participants to change their daily activities, nor their diet during study participation. No changes were made to their usual way of living, except for the start of the exercise intervention.

### RNA-seq

The total RNA from muscle samples collected from dialysis patients (at baseline and 12 months after resistance-based muscle therapy) was isolated using the TRIzol method. These RNA samples were used to prepare an mRNA library (PolyA enrichment) and were subjected to bulk mRNA sequencing on a NovaSeq X Plus Series (PE150) sequencing platform. RNA-seq data were analyzed and curated at the NovoMagic platform. RNA data submitted to the GEO repository (accession number for GEO submission is #GSE309619).

### Immunohistochemistry and immunofluorescence

Muscle tissues from dialysis patients (at baseline and 12 months after resistance-based muscle therapy) were fixed in 10% neutral buffered formalin, and paraffin-embedded tissue blocks were prepared. These blocks were then sectioned to a thickness of 5 μm. After that, tissue sections were processed for either IHC or IF staining.

For IHC, paraffin-embedded tissue sections were deparaffinized in xylene, rehydrated through a decreasing alcohol gradient (100% to 70%), and the antigenic epitope was retrieved using sodium citrate buffer (pH 6.0) in a decloaking chamber at 95 °C for 10 min. The endogenous peroxidase activity was quenched by incubating the tissue sections in BLOXALL (Vector Laboratories), followed by blocking with 5% either goat serum or horse serum according to the primary antibodies’ source/isotype for 1 h. Subsequently, the slides were incubated with primary antibodies (Supplementary Table S1) overnight in 1X antibody diluent (Epredia) at 4 °C. Next day, the slides were washed with PBST (PBS containing 0.1% Triton X), incubated with the respective secondary antibodies conjugated with HRP (Supplementary Table S1), and developed using 3,3’-Diaminobenzidine (DAB) staining (Vector Laboratories). The nuclei were counterstained with Harris Hematoxylin (Sigma-Aldrich) and then mounted in acrytol mounting medium (Leica). Images were captured using NIS-Elements imaging software in a Nikon Eclipse Ti Microscope at 40X magnification.

For IF analysis, tissue sections were incubated overnight at 4 °C with primary antibodies (See Supplementary Table S1). The next day, the slides were washed with 1X PBS and subsequently incubated with Alexa Fluor-conjugated secondary antibodies (Supplementary Table S1) for 2 h at room temperature. Slides were washed with 1X PBS. The nuclei were stained with DAPI solution (Invitrogen) and mounted with prolonged gold mounting media (Invitrogen). Images were captured using NIS-Elements imaging software on a Nikon Eclipse Ti Microscope at a magnification of 40X.

IHC and IF images were quantified by using ImageJ Fiji software NIH (Schneider et al. 2012). IHC images were quantified according to the method reported earlier (Crowe and Yue 2019), and the mean grey value represents the quantified signals and is mentioned in the results as “mean intensity”. For the quantification of IF images, the images were split into different channels, and MFI (mentioned in the results as “mean intensity”) was measured according to the protocol reported earlier (Shihan et al. 2021).

### Statistical analyses

Data was analyzed by Student’s T-test (unpaired, two-tailed) to compare the two groups using GraphPad Prism software (Version 5.03). The differences were considered significant when *p*-values were < 0.05.

## Results

### The customized muscle-based exercise upregulates the expression of genes involved in muscle generation, differentiation, and energy-generating pathways

**Fig. 1:**
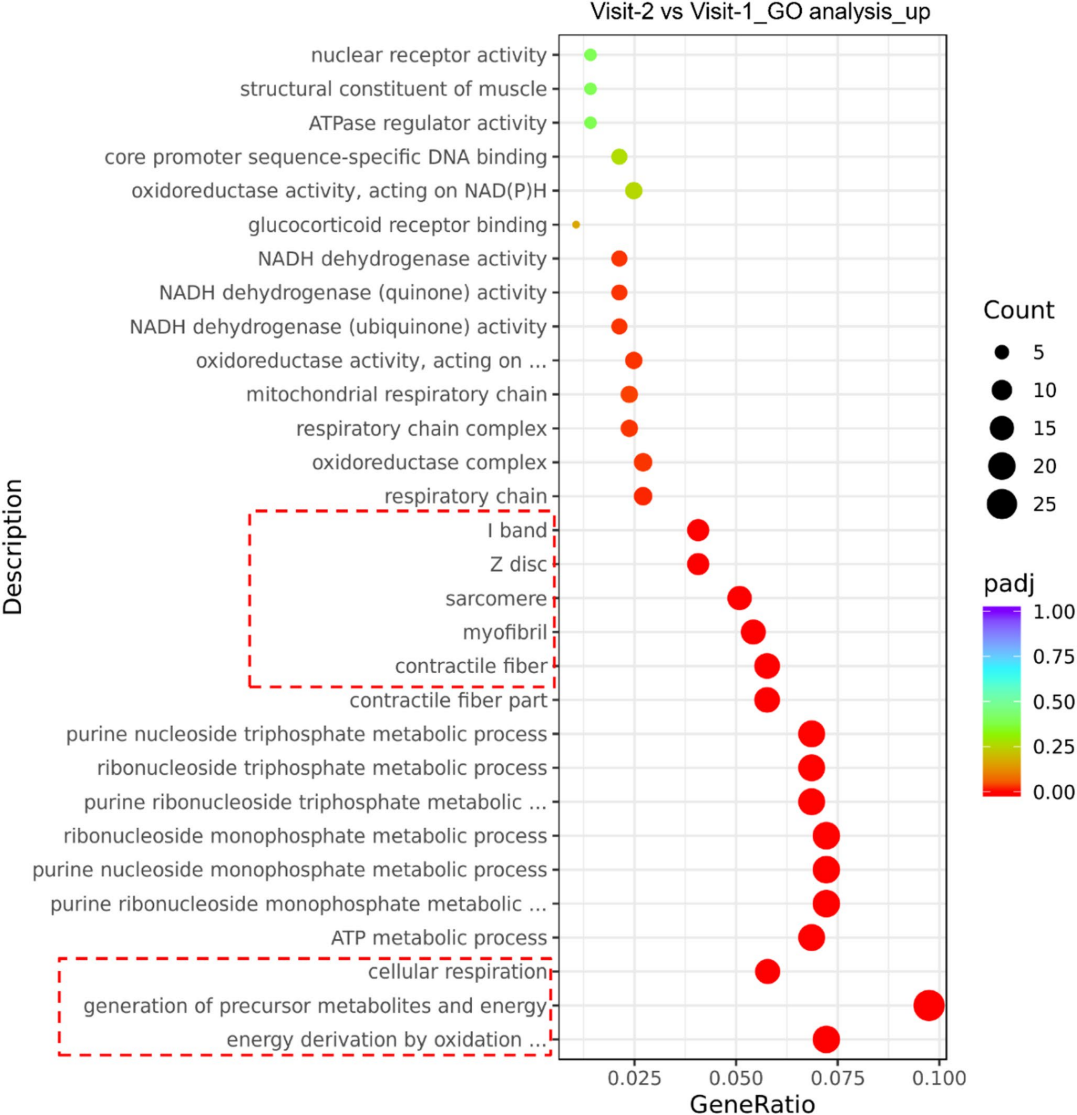
Exercise promotes gene expression of muscle generation, differentiation, and energy synthesis pathways. Gene Ontology (GO) analysis for upregulated pathways using RNA-seq data of muscle samples at basal and 12 months after exercise (*n* = 5)

To understand the modulation of various gene signatures in pre- and post-exercise muscles from dialysis patients, we performed a Gene Ontology (GO) analysis using RNA-Seq data at the NovoMagic platform. The genes involved in muscle regeneration, differentiation, and energy-generating pathways were upregulated in post-exercise muscles **(**Fig. 1 and Supplementary Fig. S1). Moreover, the expression of genes, associated with pathways involved in I-band, Z-disc, sarcomere, myofibril, and contractile fiber synthesis were upregulated in muscle samples of dialysis patients after 12 months of exercise (Fig. 1). The gene descriptions discussed here are marked in red-dotted rectangle in Fig. 1. Furthermore, the expressions of genes associated with energy supply to muscles, including cellular respiration pathway, generation of precursor metabolites and energy derivation by oxidation of organic compounds in muscle samples were increased in muscles after 12 months of exercise (Fig. 1). These results suggest that the personalized muscle exercise improves the molecular signatures of muscle regeneration, differentiation, and energy biosynthesis.

### Muscle-based exercise promotes stemness markers in the muscles of dialysis patients

**Fig. 2:**
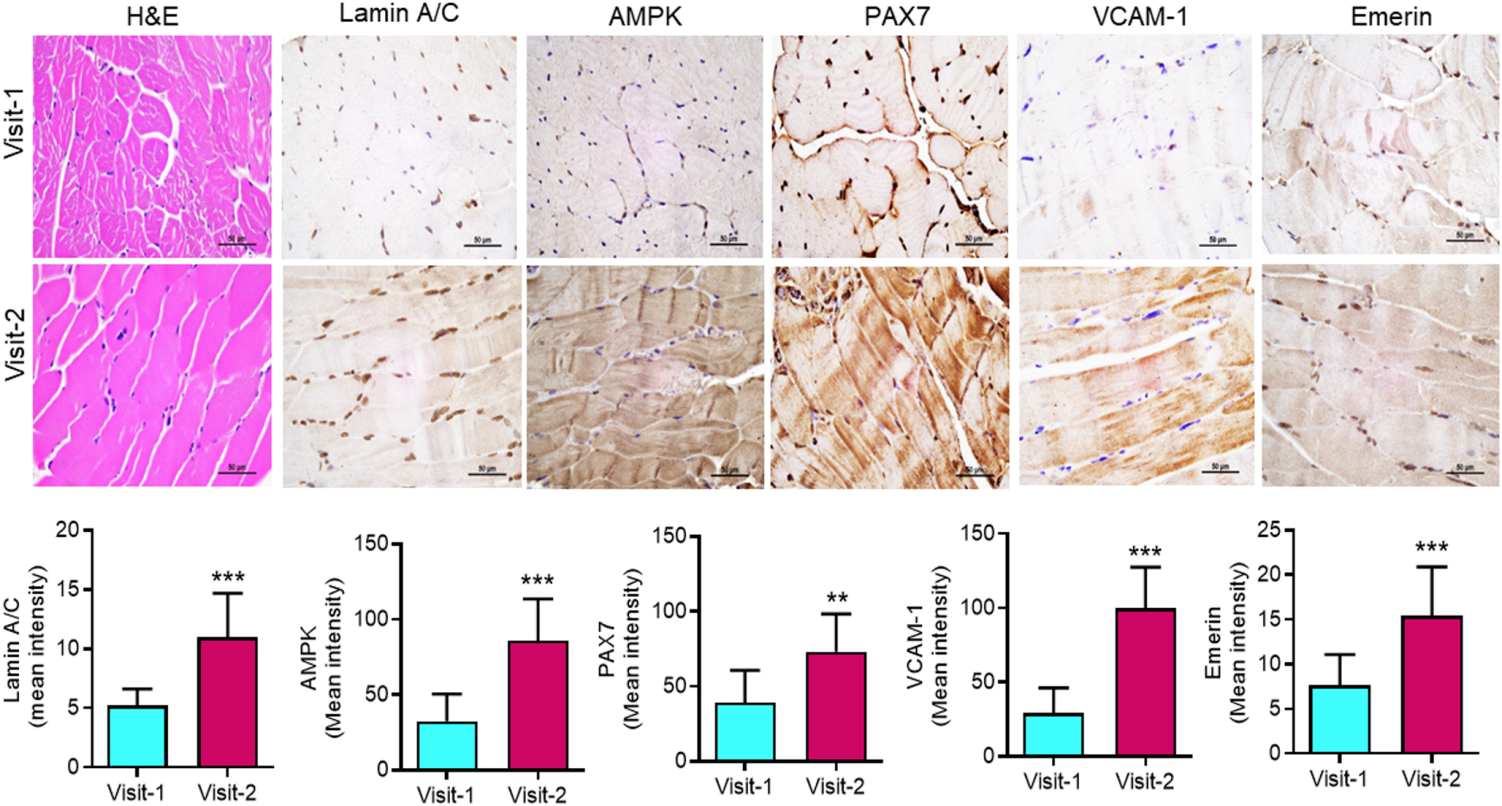
Exercise promotes stemness markers in the muscles of dialysis patients. Representative bright field images (Patient: GHD58) of hematoxylin and eosin (H&E) staining, immunohistochemistry (IHC) images (upper panel), and IHC score (lower panel) for Lamin A/C, AMPK, PAX7, VCAM-1, and Emerin of patient muscle samples for Visit-1 and Visit-2. The scale bar is 50 μm. Data are presented as mean ± SD. **p < 0.001 and ***<0.001. Student’s T-test

For self-renewal and regeneration, the muscle stemness plays a crucial role (Garcia-Prat et al. 2016). The number of muscle stem cells declines due to age and other health conditions (Garcia-Prat et al. 2016). Several proteins are known to play a critical role in maintaining muscle stemness, including Lamin A/C, adenosine monophosphate-activated protein kinase (AMPK), paired box 7 (PAX7), vascular cell adhesion molecule 1 (VCAM-1), and Emerin (Frock et al. 2006; White et al. 2018). Lamin A/C plays a crucial role in maintaining muscle stemness by regulating the differentiation of skeletal muscle satellite cells (Frock et al. 2006). AMPK regulates the balance between autophagy and apoptosis in muscles, especially in aged muscles (White et al. 2018). PAX7, a transcription factor, regulates muscle stem cell function and also plays a crucial role in muscle differentiation (Rahman et al. 2023). VCAM-1 is expressed in both inactive and active stem cells and is essential for muscle repair (Choo et al. 2017). Emerin is encoded by the EMD gene and is known for its role in muscle development (Berk et al. 2013). Considering the importance of these proteins in muscle stemness, we determined the protein expression of Lamin A/C, AMPK, PAX7, VCAM-1, and Emerin in muscles at baseline and after 12 months of exercise. The IHC analysis showed increased expression of Lamin A/C, AMPK, PAX7, VCAM-1, and Emerin in muscles after 12 months of exercise **(**Fig. 2 and Supplementary Fig. S2). These results suggest that exercise may help restore or increase the protein expression of muscle stem cell markers, which could potentially contribute to improved muscle regeneration in dialysis patients.

### Exercise induces the expression of muscle differentiation marker proteins in dialysis patients

**Fig. 3:**
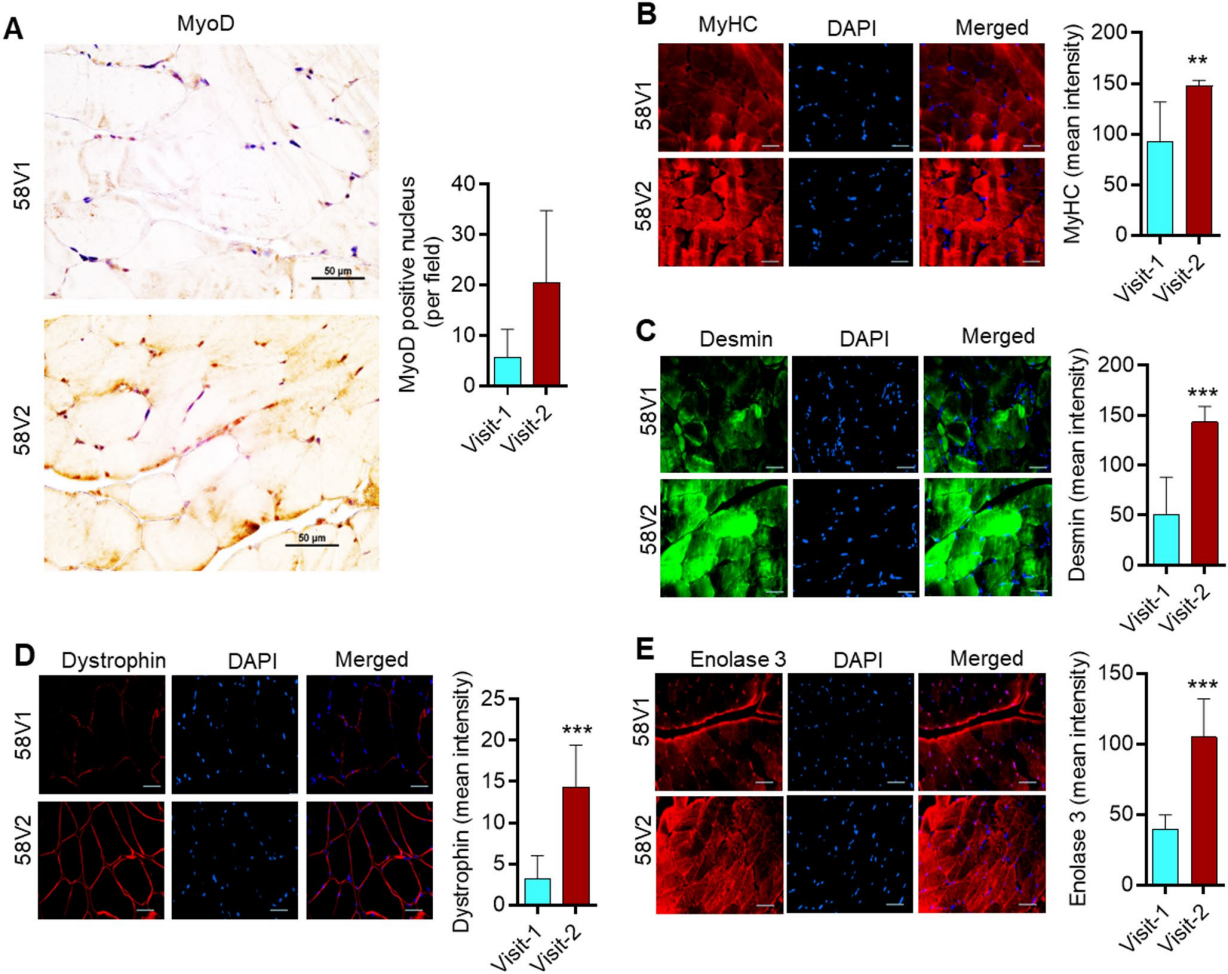
Exercise promotes expression of muscle differentiation marker proteins in dialysis patients. Representative IHC and immunofluorescence images (Patient: GHD58) and score (lower panel) for MyoD, MyHC, Desmin, Dystrophin, and Enolase 3 in patient muscle samples for Visit-1 and Visit-2. The scale bar is 50 μm. Data are presented as mean ± SD. *p < 0.05, **p < 0.001 and ***<0.001. Student’s T-test

We observed increased expression of proteins associated with muscle stemness and regeneration in patients after 12 months of exercise **(**Fig. 2**)**. Therefore, we sought to examine the status of the proteins related to muscle differentiation, including myoblast determination protein 1 (MyoD), myosin heavy chain (MyHC), desmin, dystrophin, and enolase 3. Master regulator of muscle differentiation, MyoD, is a transcription factor that plays a critical role in muscle differentiation (Wu and Yue 2024). MyHC is known to regulate muscle fiber size, number, and other parameters, as well as muscle differentiation (Agarwal et al. 2020; Zammit 2017). Muscle-specific intermediate filament protein desmin plays an essential role in muscle maturity and homeostasis (Li et al. 1997). Like desmin, another protein, dystrophin is necessary for both muscle differentiation and muscle integrity (Gosselin et al. 2022). Beta enolase 3 (enolase 3) is a crucial protein known for its role in muscle differentiation and maturity (Fougerousse et al. 2001). We examined the protein expression of MyoD, MyHC, desmin, dystrophin, and enolase 3 in muscles at baseline and after 12 months of exercise. The IF analysis revealed increased expression of MyoD, MyHC, desmin, dystrophin, and enolase 3 in muscles after 12 months of exercise compared to the baseline **(**Fig. 3 and Supplementary Fig. S3). These results suggest that personalized exercise therapy increases the expression of muscle-differentiating proteins, which could potentially contribute to improved muscle differentiation, alignment, and integrity in dialysis patients.

### Exercise promotes gene and protein expressions of markers for healthy mitochondria in the muscles of dialysis patients

**Fig. 4:**
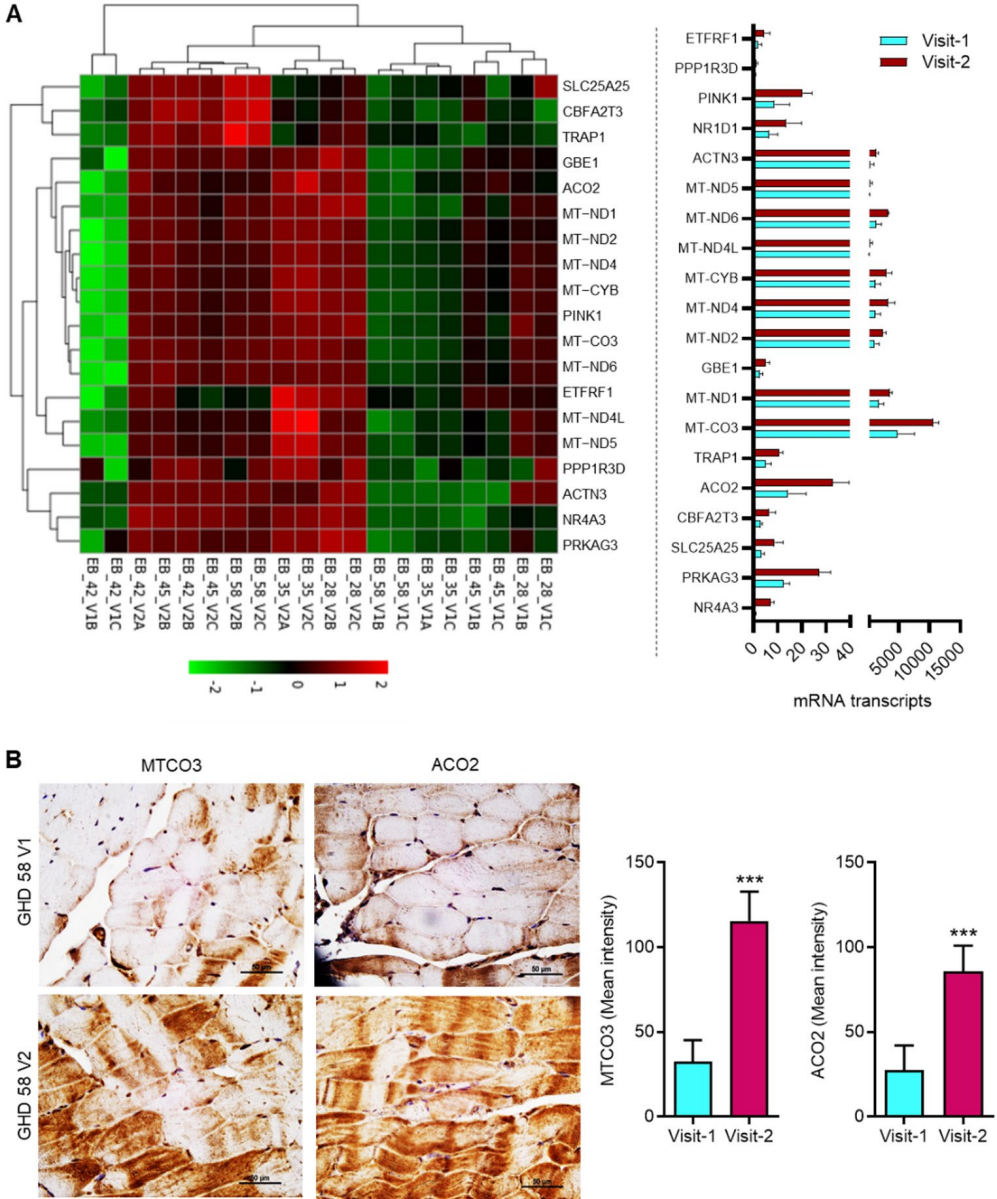
Exercise promotes gene and protein expressions of markers for healthy mitochondria in the muscles of dialysis patients. **(A)** RNA-seq data from muscle samples at baseline and 12 months after exercise were analyzed for gene expression related to energy derivation through oxidation pathways (*n* = 5). **(B)** Representative IHC images (Patient: GHD58) and score for MTCO3 and ACO2 in patient muscle samples for Visit-1 and Visit-2. The scale bar is 50 μm. Data are presented as mean ± SD. ***p < 0.001. Student’s T-test. EB_28_V1: Patient GHD28-Visit-1; EB_28_V2: Patient GHD28-Visit-2; EB_35_V1: Patient GHD35-Visit-1; EB_35_V2: Patient GHD35-Visit-2; EB_42_V1: Patient GHD42-Visit-1; EB_42_V2: Patient GHD42-Visit-2; EB_45_V1: Patient GHD45-Visit-1; EB_45_V2: Patient GHD45-Visit-2; EB_58_V1: Patient GHD58-Visit-1; EB_58_V2: Patient GHD58-Visit-2

Mitochondria play an essential role in muscle function (Brand et al. 2013). Based on transmission electron microscopy (TEM) analysis, we reported that exercise enhances mitochondrial count and promotes healthy mitochondrial morphology (Bartlett et al. 2025). We determined the protein expression of COX-IV and ATP5I in muscle tissue at the baseline and 12 months after exercise. IF analysis showed increased expression of COX-IV and ATP5I in the muscle from dialysis patients after 12 months of exercise **(Supplementary Fig. S4)**. Since RNA-seq analysis revealed upregulation of pathway associated with oxidation of organic compounds for energy generation **(**Fig. 1**)**, we determined expressions of various genes involved in oxidation of organic compounds for energy generation including, *NR4A3*,* PRKAG3*,* SLC25A25*,* CBFA2T3*,* ACO2*,* TRAP1*,* MT-CO3*,* MT-ND1*,* GBE1*,* MT-ND2*,* MT-ND4*,* MT-CYB*,* MT-ND4L*,* MT-ND6*,* MT-ND5*,* ACTN3*,* NR1D1*,* PINK1*,* PPP1R3D*,* and ETFRF1* **(**Fig. 4A**)**. Moreover, the protein expressions of two highly altered genes including mitochondrial encoded cytochrome c oxidase III (MTCO3) and aconitase 2 (ACO2), were determined in muscles by IHC analysis. The exercise therapy increased the protein expression of MTCO3 and ACO2 in the muscles of dialysis patients after 12 months of exercise **(**Fig. 4B and Supplementary Fig. S4). These results suggest that exercise increases the gene and protein expression of MTCO3 and ACO2 in muscle, which could potentially contribute to improved muscle energy generation by oxidation of organic compounds in dialysis patients.

### Exercise promotes the expression of key proteins involved in energy generation through the utilization of glucose, glycogen, and fatty acids

**Fig. 5:**
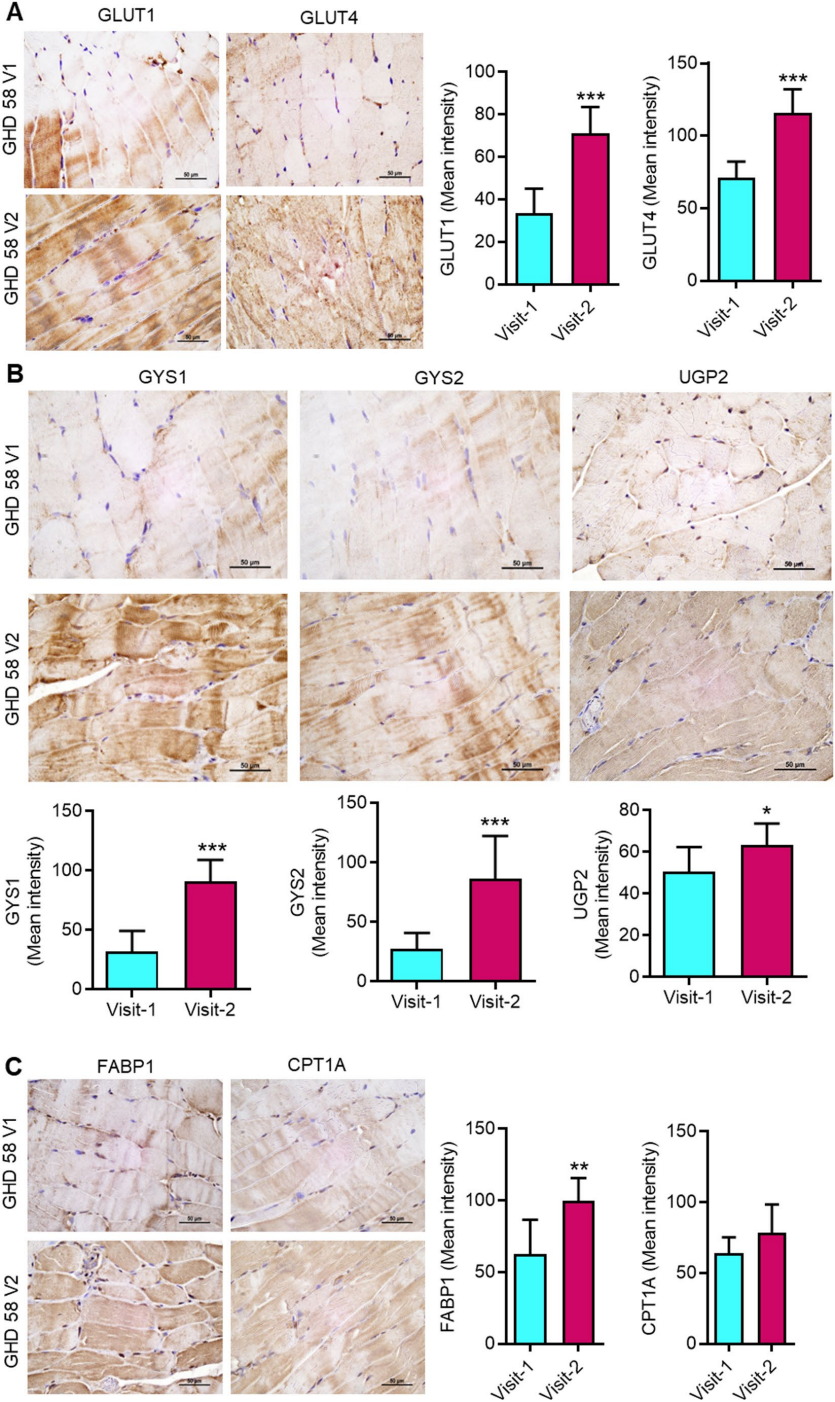
Exercise promotes protein expression of key enzymes involved in energy generation via oxidation of organic compounds. **(A)** Representative IHC images (Patient: GHD58) and score for GLUT1 and GLUT4 in patient muscle samples for Visit-1 and Visit-2. **(B)** Representative IHC images (Patient: GHD58) and score for GYS1, GYS2, and UGP2 in patient muscle samples for Visit-1 and Visit-2. **(C)** Representative IHC images (Patient: GHD58) and score for FABP1 and CPT1A in patient muscle samples for Visit-1 and Visit-2. The scale bar is 50 μm. Data are presented as mean ± SD. ***p < 0.001. Student’s T-test

The RNA-seq analysis showed increased expression of genes associated with energy generation pathways, including those facilitated by organic compounds (Shadel and Horvath 2015). The glucose uptake, glycogenesis, and fatty acid metabolism-induced energy generation are essential for muscle function (Shadel and Horvath 2015). The glucose uptake mediated by glucose transporter protein types 1 (GLUT1) and 4 (GLUT4) plays a crucial role in muscle function (Buse et al. 1996). We determined the protein expression of GLUT1 and GLUT4 in muscle samples at the baseline and 12 months after exercise. There was increased expression of GLUT1 and GLUT4 proteins in muscle samples after 12 months of exercise **(**Fig. 5A and Supplementary Fig. S5A). These results suggest that exercise increases the protein expression of GLUT1 and GLUT4 in muscle of dialysis patients, which could potentially contribute to improved glucose uptake and energy generation. Glycogen synthase (GYS) members, GYS1 and GYS2, are rate-limiting enzymes in glycogen biosynthesis (De Filippi et al. 2023). Additionally, UDP-glucose pyrophosphorylase 2 (UGP2) is essential for the biosynthesis of glycogen precursor (Perenthaler et al. 2020). We determined the protein expression levels of GYS1, GYS2, and UGP2 in muscle samples at baseline and 12 months after exercise. IHC analysis revealed that exercise enhances the expression of GYS1, GYS2, and UGP2 proteins in muscles of dialysis patients **(**Fig. 5B and Supplementary Fig. S5B). These findings suggest that exercise could potentially contribute to improved glycogen-mediated energy generation in the muscles of dialysis patients. Furthermore, we determined the levels of key proteins, including FABP1 (fatty acid-binding protein 1) and CPT1A (carnitine palmitoyl transferase 1 A), which are known to regulate fatty acid metabolism for energy generation in muscle (Hammoud et al. 2024; Schroeder et al. 2016). IHC analysis revealed that exercise increases expression of FABP1 protein in dialysis patients **(**Fig. 5C and Supplementary Fig. S5C). These results suggest that the exercise therapy augments the protein expression of FABP1 in muscle, which could potentially contribute to improved energy generation by fatty acids in dialysis patients.

## Discussion

Loss of muscle mass and strength is a major health concern in dialysis patients. Some studies have demonstrated that exercise could prevent or restore muscle mass and strength through promoting muscle differentiation (Krzysztofik et al. 2019; Konopka and Harber 2014). Previous studies have suggested that an optimal practice of physical training may lead to better outcomes in dialysis patients (Romano et al. 2012). It has been reported that exercise training for dialysis patients is a practical approach to improving physical capacity and quality of life (De Smet and Van Craenenbroeck 2021). Earlier, we reported that personalized exercise therapy improves muscle health in patients undergoing dialysis (Bartlett et al. 2025). To further understand the underlying mechanisms by which our personalized exercise therapy improves muscle strength (Bartlett et al. 2025) in dialysis patients, RNA-Seq analysis was conducted, revealing that exercise promotes gene signatures related to muscle generation, differentiation, and energy synthesis.

Muscle stemness plays a crucial role in the self-renewal and regeneration of muscles (Garcia-Prat et al. 2016). The stem cell associated with skeletal muscle is known as satellite cells (Abreu and Kowaltowski 2020). Several proteins have been studied, including Lamin A/C, AMPK, PAX7, VCAM-1, and Emerin, that play crucial functional roles in muscle stemness (Frock et al. 2006; White et al. 2018; Rahman et al. 2023; Choo et al. 2017; Berk et al. 2013). It has been reported that endurance and resistance-based muscle training can lead to muscle stemness, which facilitates muscle renewal and regeneration (Liu et al. 2023; Abreu and Kowaltowski 2020). The effect of excersize has been studied on the AMPK signaling (Chen et al. 2003). For example, men cycling for 20 min with three different sequential intensity showed that muscle free AMP/ATP ratio was found increased in higher intensity excersize (Chen et al. 2003). Previously, a study conducted to investigate change in PAX7 expression in response to resistance exercise in young men that showed significant increase in PAX7 protein expression in the muscle (Karimi Majd et al. 2023). Based on published literature and our results, we anticipate that personalized exercise therapy may enhance the muscle stem cell phenotypes, thereby contributing to muscle regeneration.

Muscle differentiation is a process that covers a broad range of muscle activities, including growth, repair, endurance, adaptation, and overall muscle health (Jalal et al. 2021). The process of myogenic differentiation can be easily traced by determining myogenic differentiation markers, such as PAX7, MyoD, and myosin heavy chain isoforms (Hoseini et al. 2025; Wu and Yue 2024; Agarwal et al. 2020; Zammit 2017; Li et al. 1997; Gosselin et al. 2022; Fougerousse et al. 2001). The increased expression of MyoD, MyHC, desmin, dystrophin, and enolase 3 proteins in post-exercise muscles clearly suggests that our personalized exercise therapy restores the myogenic differentiation pathways, thereby enhancing the observed muscle strength in dialysis patients. In a mouse model of exercise, it has been reported that exercise promotes MyoD mRNA stability in skeleton muscles in a Mettl3-mediated m6A-dependent manner (Feng et al. 2024). Like MyoD the increased protein expression has also been reported for MyHC in a murine model of muscle training exercise (Demirel et al. 1999). In study associated with high intensity cycling training in men showed highly increased protein content of desmin that was correlated with enhanced muscular adaptation (Woolstenhulme et al. 2005). Like desmin, the protein expression of dystrophin is also involved in muscular adoptaion mainly by stabilizing cytoskeletan and prevent cells against contraction-induced abnormalities (Kaplan and Morgan 2022). Our results clearly support the notion that personalized exercise therapy increases muscle-differentiating markers, indicating improved muscle differentiation, alignment, and integrity in patients undergoing dialysis.

Mitochondria, also known as the powerhouse of cells, play an essential role in skeletal muscle function (Brand et al. 2013). In our recent report, we reported that our personalized exercise therapy increased the mitochondrial count and healthy mitochondrial morphology (Bartlett et al. 2025). The RNA-seq analysis of muscle samples revealed increased expression of genes involved in mitochondrial biosynthesis and function. The MTCO3 is known to play an essential role in the mitochondrial respiratory chain by regulating cytochrome c oxidase subunit II (Mkaouar-Rebai et al. 2011). The MTCO3-regulated electron transport chain (ETC) generates most of the energy through ATP generation and functional role of MTCO3 in relation to exercise has already been published in men (Zhou et al. 2025). ACO2 is crucial for the TCA cycle in mitochondria (You et al. 2021) and catalyzes the conversion of citrate to isocitrate, a vital step in cellular energy production (You et al. 2021). Privious report suggests that exercise increases ACO2 expression and mitochondrial biogenesis (Chen et al. 2020). Furthermore, increase in ACO2 expression post exercise reflects enhanced oxidative energy metabolism that support post-exercise recovery and ATP demand (Fukawa et al. 2025).

Cellular energy generation requires raw materials, including organic compounds like carbohydrates, proteins, and lipids (Shadel and Horvath 2015). The importance of carbohydrates, proteins, and lipids in facilitating energy generation during exercise is well known and reiterated by several groups (Pi et al. 2023; Grevendonk et al. 2021). The role of glucose transporters is critical for glucose uptake by muscles (Klip and Paquet 1990). In the present study, the increased protein expression of glucose transporters (GLUT1 and GLUT4) highlights the significant role of exercise in regulating glucose metabolism for muscle function. A preclinical study to conducted to observe the influence of physical exercise on the expression of GLUT 1 in muscle tissue showed that GLUT 1 expressions were significantly higher in the exercising groups (Heled et al. 2005). A endurance training conducted in healthy men showed significan increase in GLUT-1 and GLUT-4 protein expression in skeletal muscle (Phillips et al. 1996). Glycogen is a stored form of glucose, mainly in the liver and muscles (Kanungo et al. 2018). The rate-limiting enzymes GYS1 and GYS2, as well as the glycosylation enzyme UGP2, play a crucial role in glycogen biosynthesis (De Filippi et al. 2023; Perenthaler et al. 2020). In present study, the increased protein expression of GYS1, GYS2, and UGP2 suggests a significant role of exercise in regulating glycogen metabolism and supporting muscle strength and function in dialysis patients. Similar to glucose and glycogen, fatty acids are an important source of energy for muscles during exercise (Fritzen et al. 2020). FABP1 and CPT1A are key regulatory proteins involved in the regulation of fatty acid metabolism for energy synthesis in muscle during exercise (Jayewardene et al. 2016). The increased protein expression of FABP1 in post-exercise muscles establishes a significant role of exercise in regulating fatty acid metabolism for energy generation. These results suggest that personalized exercise improves glucose, glycogen, and fatty acid metabolism in skeletal muscle, which could potentially contribute to enhanced muscle function through improved energy generation in dialysis patients.

## Conclusions

**Fig. 6:**
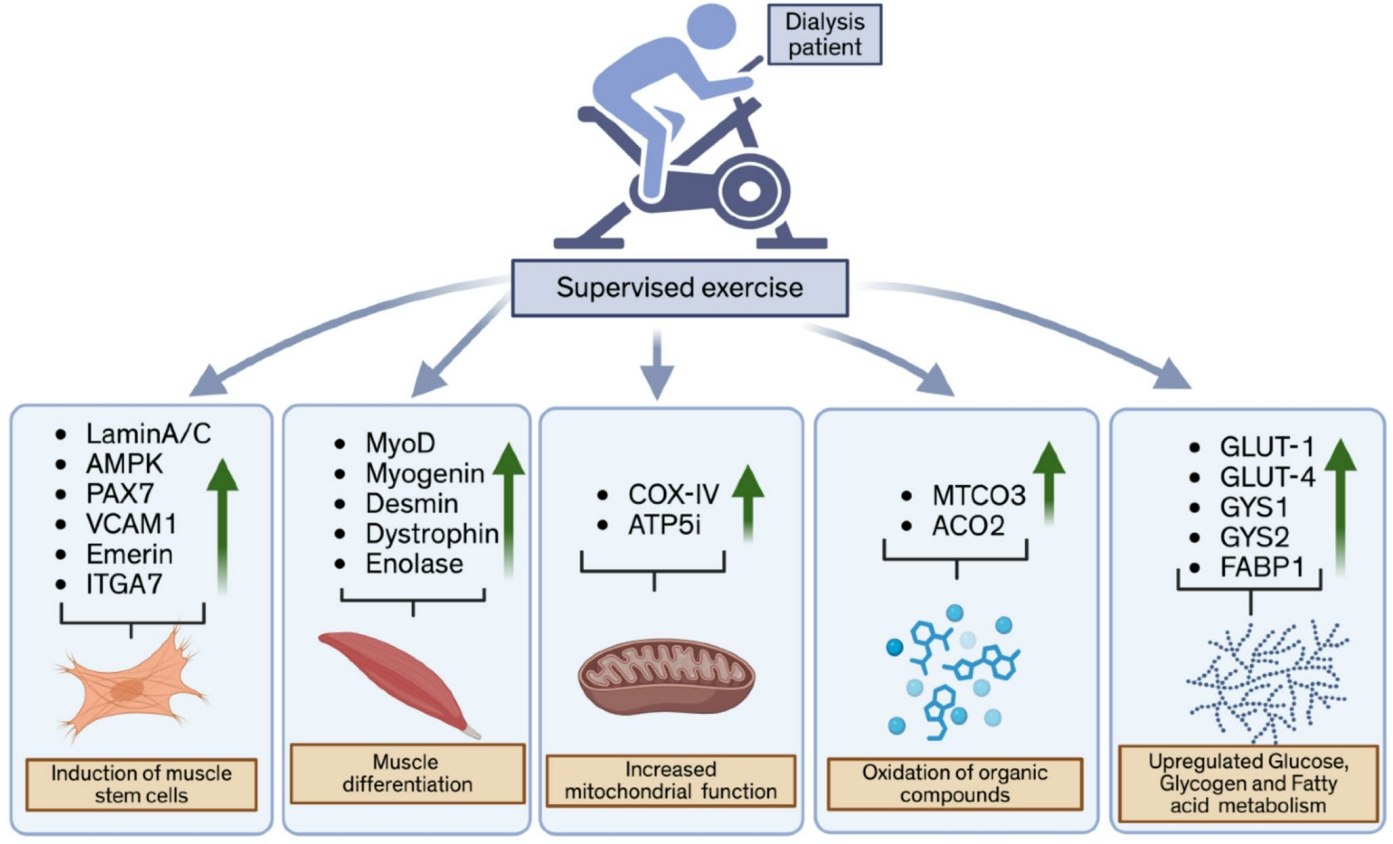
Schematic diagram showing the impact of muscle exercise on dialysis patients. Based on our findings, exercise can induce muscle stemness, regeneration, differentiation, and muscle functions

To understand the status of gene signatures of various pathways important for muscle health, we performed RNA-seq analysis in muscle samples obtained from resistance-based muscle therapy in dialysis patients. Our results clearly support the notion that our personalized exercise therapy enhances the muscle stem cell phenotypes that contribute to muscle regeneration. Moreover, we observed that exercise increased the expression of muscle-differentiating markers, suggesting improved muscle differentiation, alignment, and integrity in patients undergoing dialysis. Furthermore, exercise increases MTCO3 and ACO2, which are associated with the oxidation of organic compounds. However, the exact role of exercise in relation to increased MTCO3 and ACO2 expression and function requires further exploration. Our results indicate that exercise enhances glucose, glycogen, and fatty acid metabolism pathways in skeletal muscle, which could potentially contribute to improved muscle energy generation in dialysis patients. In summary, the in-depth molecular analyses of patients’ muscles after the personalized exercise therapy support the improved muscle health of dialysis patients and should be implemented as a complementary therapy after transplantation **(**Fig. 6).

## Supplementary Information

Below is the link to the electronic supplementary material.

Supplementary Material 1

## Data Availability

The datasets generated and/or analyzed during the current study are available in the NCBI’s Gene Expression Omnibus (GEO) repository and can be accessed through accession number GSE309619.

## Abbreviations

AMPK: AMP-activated protein kinase
ATP: Adenosine triphosphate
ATP5I: ATP synthase membrane subunit E
CKD: Chronic Kidney Disease
COX-IV: Cytochrome c oxidase subunit 4
CPT1A: Carnitine Palmitoyl Transferase 1 A
DAB 3: 3’-Diaminobenzidine
Enolase 3: Beta enolase 3
ESKD: End-stage kidney disease
ETC: Electron transport chain
FABP1: Fatty acid binding protein 1
GFR: Glomerular filtration rate
GO: Gene Ontology
IF: Immunofluorescence
IHC: Immunohistochemistry
IRB: Institutional Review Board
MSE: Muscle-strengthening exercise
MyoD: Myoblast determination protein 1
MyHC: Myosin heavy chain 1 H
PAX7: Paired box 7
VCAM-1: Vascular cell adhesion molecule 1
