# Supplementary material for: Resistance-based exercise restores muscle health in dialysis patients
Source: J Muscle Res Cell Motil. 2026 Mar 14;47(1):8. doi: 10.1007/s10974-026-09727-0 (PMC12989000; doi:10.1007/s10974-026-09727-0)
Supplement: Supplementary file 1 — Supplementary Material 1 [file 10974_2026_9727_MOESM1_ESM.pdf]

Supplementary Materials

Resistance-based exercise restores muscle health in dialysis patients.

Piush Srivastava et al.,

This PDF contains:

Supplementary Table and Figure legends

Table S1

Figure S1

Figure S2

Figure S3

Figure S4

Figure S5

Supplementary material legends:

Supplementary Table 1: List of antibodies used for immunohistochemistry (IHC) and immunofluorescence (IF) staining.

Supplementary Figure 1: Gene Ontology (GO) analysis for downregulated pathways using RNA-seq data of muscle samples at basal and 12 months after exercise (n=5).

Supplementary Figure 2: Representative bright field images (Patients: GHD62 and GHD65) of hematoxylin and eosin (H&E) staining, immunohistochemistry (IHC) for Lamin A/C, AMPK, PAX7, VCAM-1, and Emerin of patient muscle samples for Visit-1 and Visit-2. The scale bar is 50 μm.

Supplementary Figure 3: Representative immunofluorescence images (Patients: GHD62 and GHD65) for MyoD, MyHC, Dystrophin, and Enolase 3 in patient muscle samples for Visit-1 and Visit-2. The scale bar is 50 μm.

Supplementary Figure 4: (A-B) Representative immunofluorescence images (Patients: GHD58, GHD62, and GHD65) for COX-IV and ATP5I, and **(C)** Representative IHC images (Patient: GHD62 and GHD65) for MTCO3 and ACO2 in patient muscle samples for Visit-1 and Visit-2. The scale bar is 50 μm. Data are presented as mean ± SD. **p<0.001. Student's t-test.

Supplementary Figure 5: (A) Representative IHC images (Patients: GHD62 and GHD65) for GLUT1 and GLUT4 in patient muscle samples for Visit-1 and Visit-2. **(B)** Representative IHC images (Patients: GHD62 and GHD65) for GYS1, GYS2, and UGP2 in patient muscle samples for Visit-1 and Visit-2. **(C)** Representative IHC images (Patients: GHD62 and GHD65) for FABP1 and CPT1A in patient muscle samples for Visit-1 and Visit-2. The scale bar is 50 μm. Data are presented as mean ± SD. ***p<0.001. Student's t-test.

Table S1: List of antibodies used for immunohistochemistry (IHC) and immunofluorescence (IF) staining

Antibody	Catalog	Company	Dilution	Application
COX IV	ab16056	Abcam	1:1000	IF
Myosin	ab37484	Abcam	1:300	IF
ATP5I	16483-1-AP	Proteintech	1:200	IF
Desmin	16520-1-AP	Proteintech	1:100	IF
MyoD1	18943-1-AP	Proteintech	1:50	IF
Dystrophin	MA5-49837	Invitrogen	1:50	IF
Enolase	55234-1-AP	Proteintech	1:50	IF
MTCO3	55082-1-AP	Proteintech	1:200	IHC
Aconitase 2	11134-1-AP	Proteintech	1:200	IHC
PAX7	20570-1-AP	Proteintech	1:200	IHC
Lamin A/C	10298-1-AP	Proteintech	1:2000	IHC
AMPK	10929-2-AP	Proteintech	1:200	IHC
VCAM1	14-1069-82	Invitrogen	1:75	IHC
GYS1	10566-1-AP	Proteintech	1:200	IHC
GYS2	22371-1-AP	Proteintech	1:200	IHC
GLUT1	21829-1-AP	Proteintech	1:1000	IHC
GLUT4	66846-1-Ig	Proteintech	1:200	IHC
CPT1A	1584-1-AP	Proteintech	1:1000	IHC
FABP1	13626-1-AP	Proteintech	1:200	IHC
UGP2	10491-1-AP	Proteintech	1:100	IHC

Emerin	10351-1-AP	Proteintech	1:1000	IHC
Goat anti-Rabbit IgG (H+L), Alexa Fluor™ 488	A-11008	Invitrogen	1:300	IF
Goat anti-Rabbit IgG (H+L), Alexa Fluor™ 595	A-11012	Invitrogen	1:300	IF
Goat anti-Mouse IgG (H+L), Alexa Fluor™ 594	A-11005	Invitrogen	1:300	IF

Figure S1

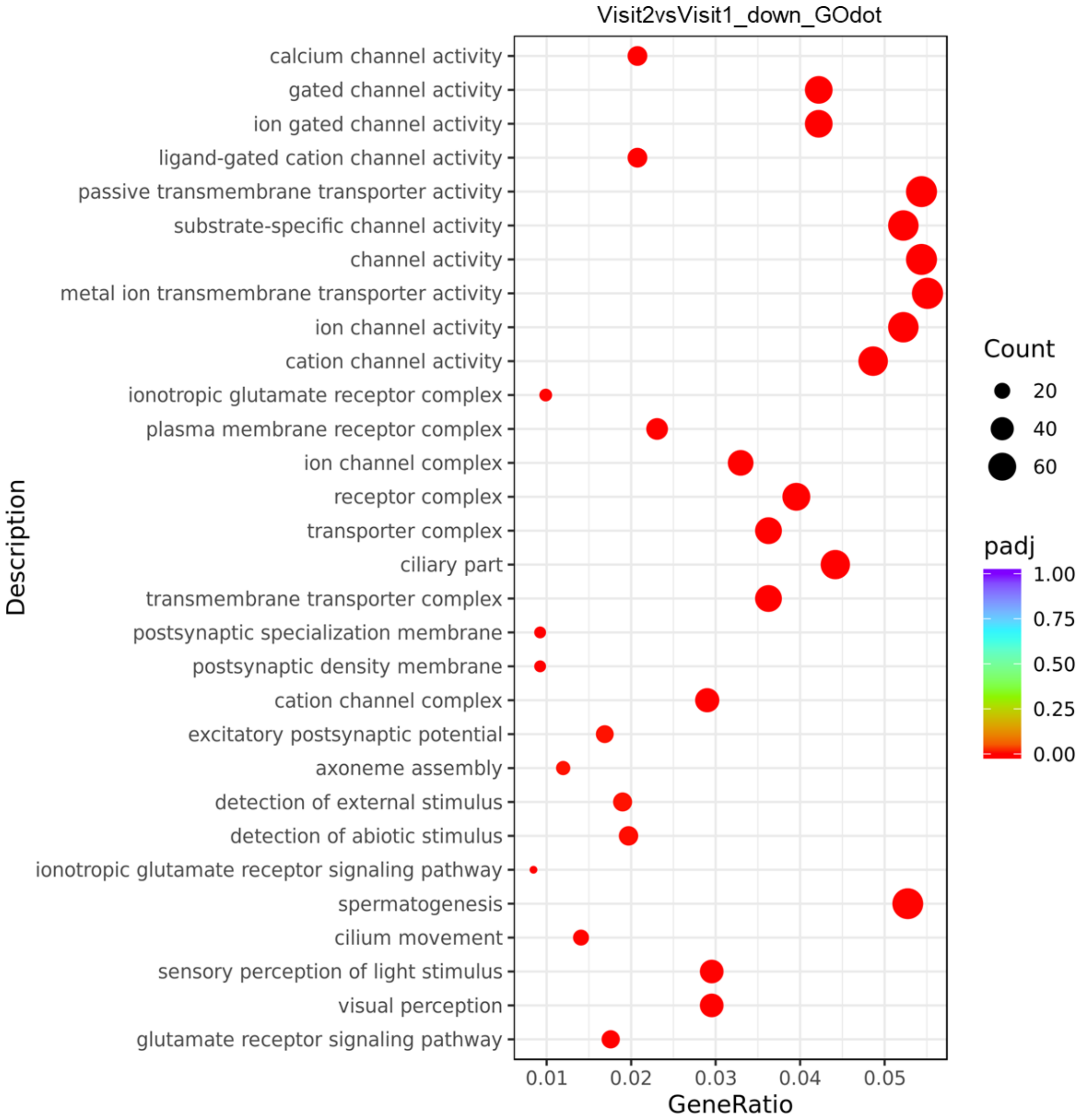

Visit2vsVisit1_down_GOdot
calcium channel activity
gated channel activity
ion gated channel activity
ligand-gated cation channel activity
passive transmembrane transporter activity
substrate-specific channel activity
channel activity
metal ion transmembrane transporter activity
ion channel activity
cation channel activity
ionotropic glutamate receptor complex
plasma membrane receptor complex
ion channel complex
receptor complex
transporter complex
ciliary part
transmembrane transporter complex
postsynaptic specialization membrane
postsynaptic density membrane
cation channel complex
excitatory postsynaptic potential
axoneme assembly
detection of external stimulus
detection of abiotic stimulus
ionotropic glutamate receptor signaling pathway
spermatogenesis
cilium movement
sensory perception of light stimulus
visual perception
glutamate receptor signaling pathway
Description
0.01
0.02
0.03
0.04
0.05
GeneRatio
Count
20
40
60
padj
1.00
0.75
0.50
0.25
0.00

Figure S2

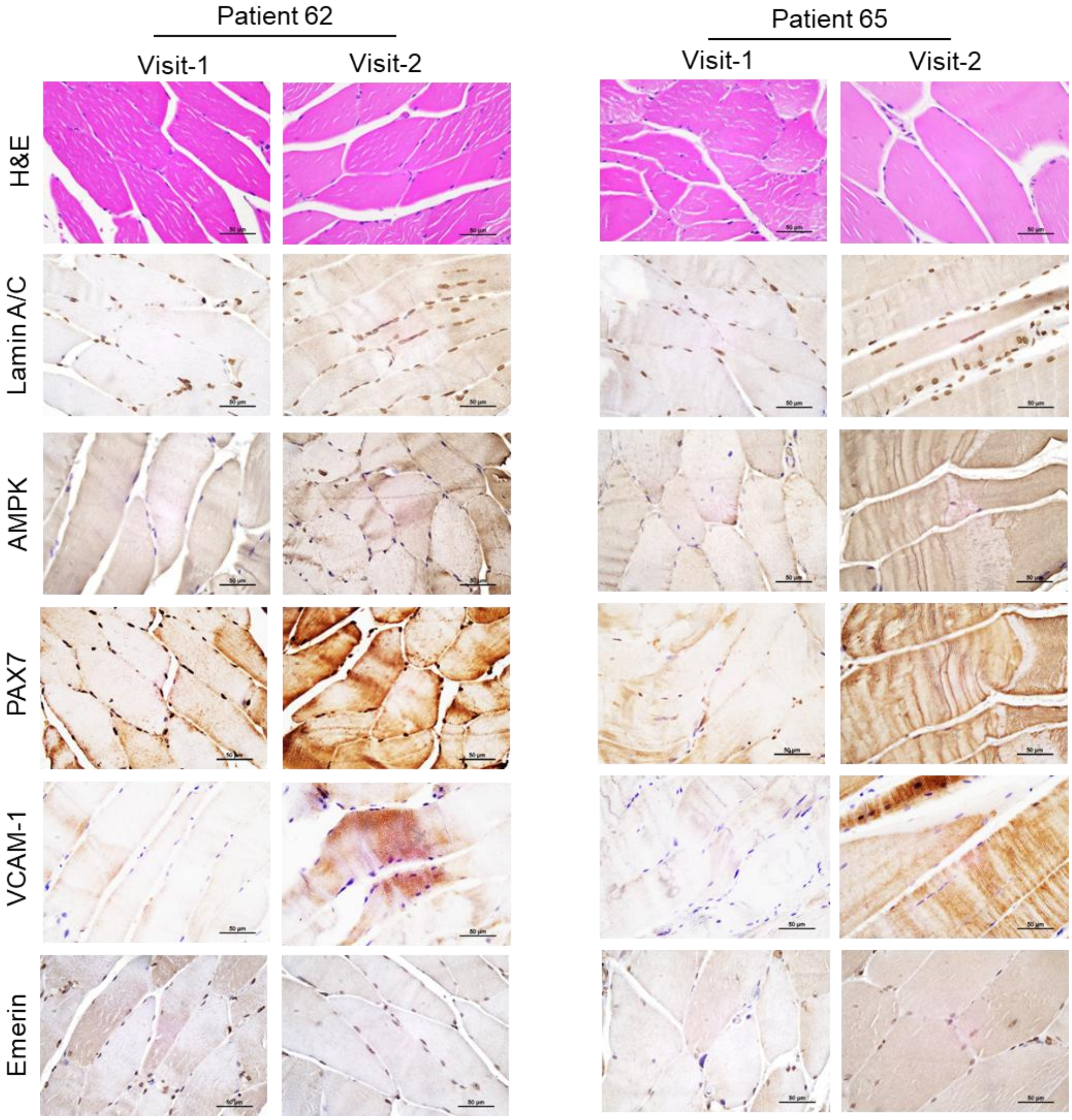
Patient 62
Patient 65
Visit-1
Visit-2
Visit-1
Visit-2
H&E
Lamin A/C
AMPK
PAX7
VCAM-1
Emerin

Figure S3

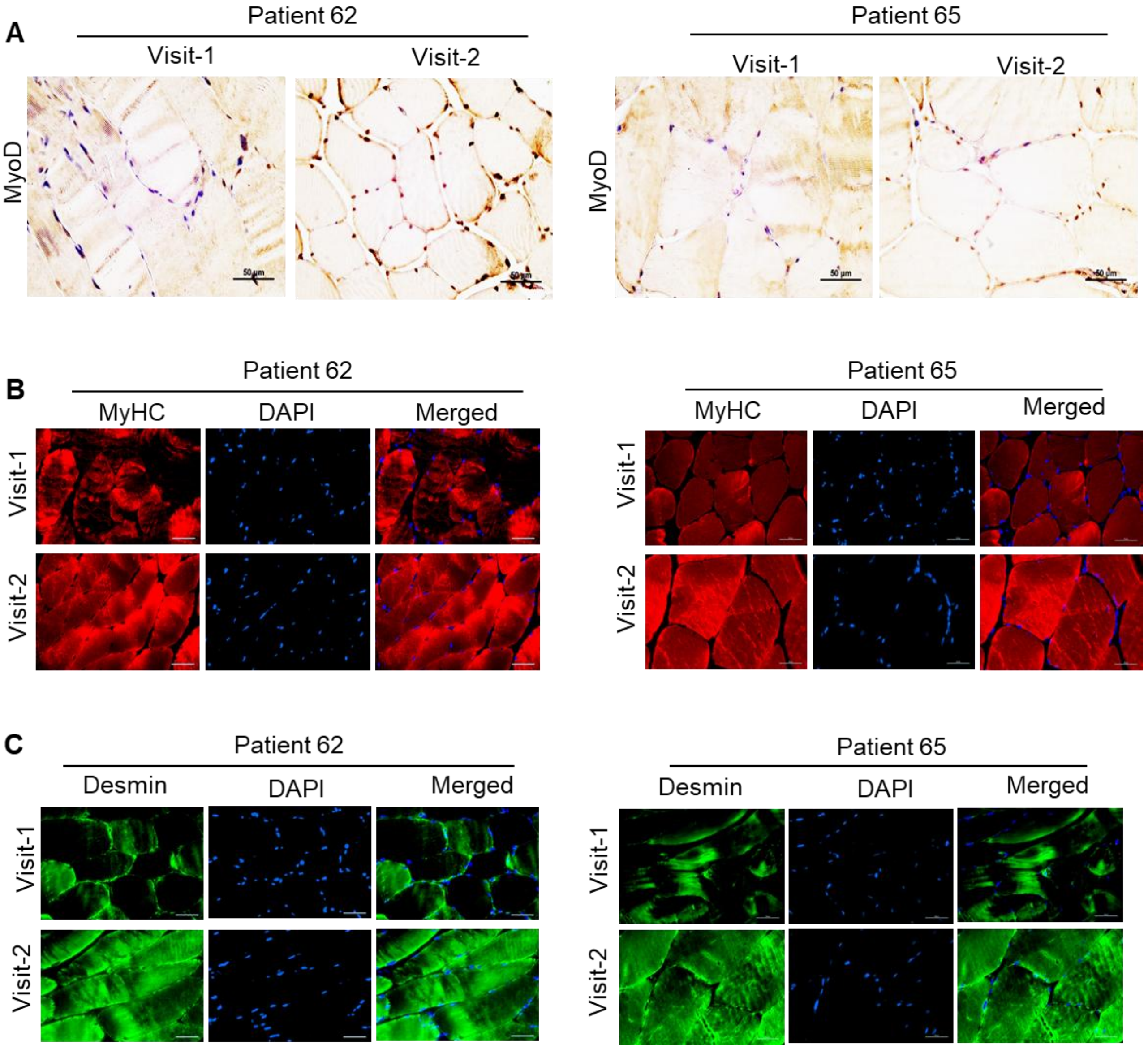
A
Patient 62
Visit-1
Visit-2
MyoD
Patient 65
Visit-1
Visit-2
MyoD
50 µm
B
Patient 62
MyHC
DAPI
Merged
Visit-1
Visit-2
Patient 65
MyHC
DAPI
Merged
Visit-1
Visit-2
C
Patient 62
Desmin
DAPI
Merged
Visit-1
Visit-2
Patient 65
Desmin
DAPI
Merged
Visit-1
Visit-2

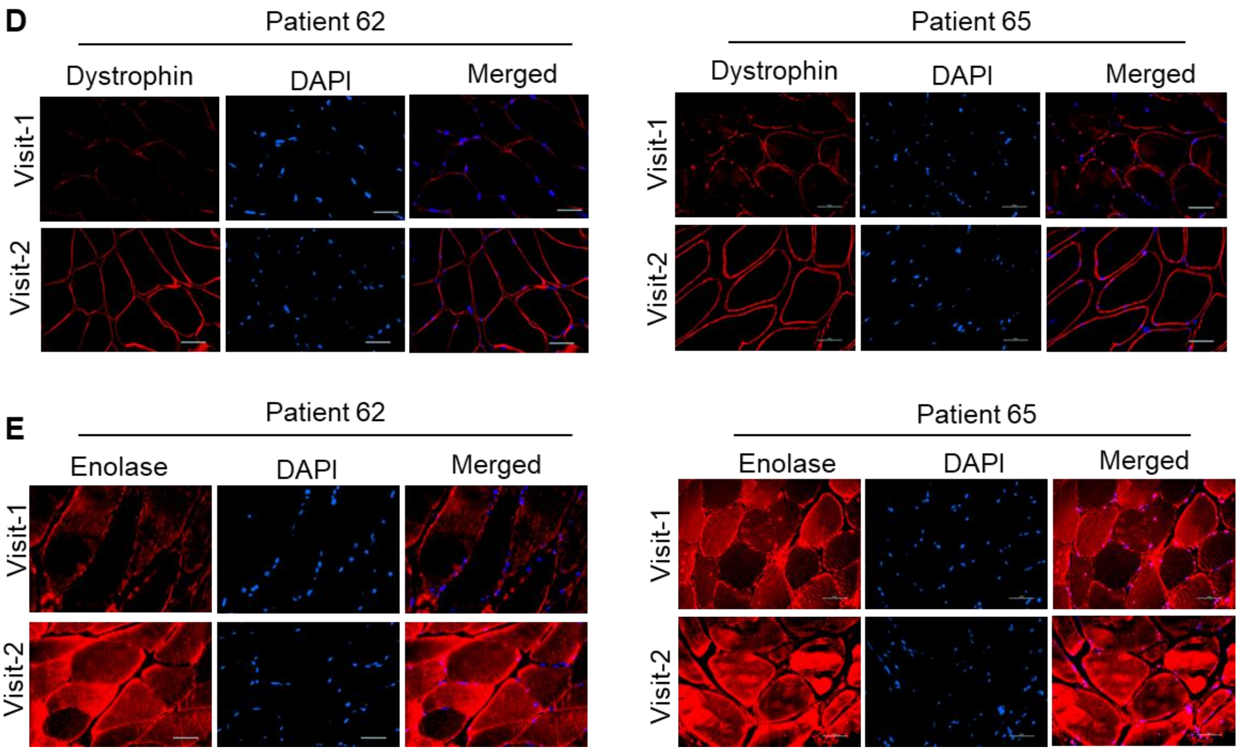
D
Patient 62
Dystrophin
DAPI
Merged
Visit-1
Visit-2
Patient 65
Dystrophin
DAPI
Merged
Visit-1
Visit-2
E
Patient 62
Enolase
DAPI
Merged
Visit-1
Visit-2
Patient 65
Enolase
DAPI
Merged
Visit-1
Visit-2

Figure S4

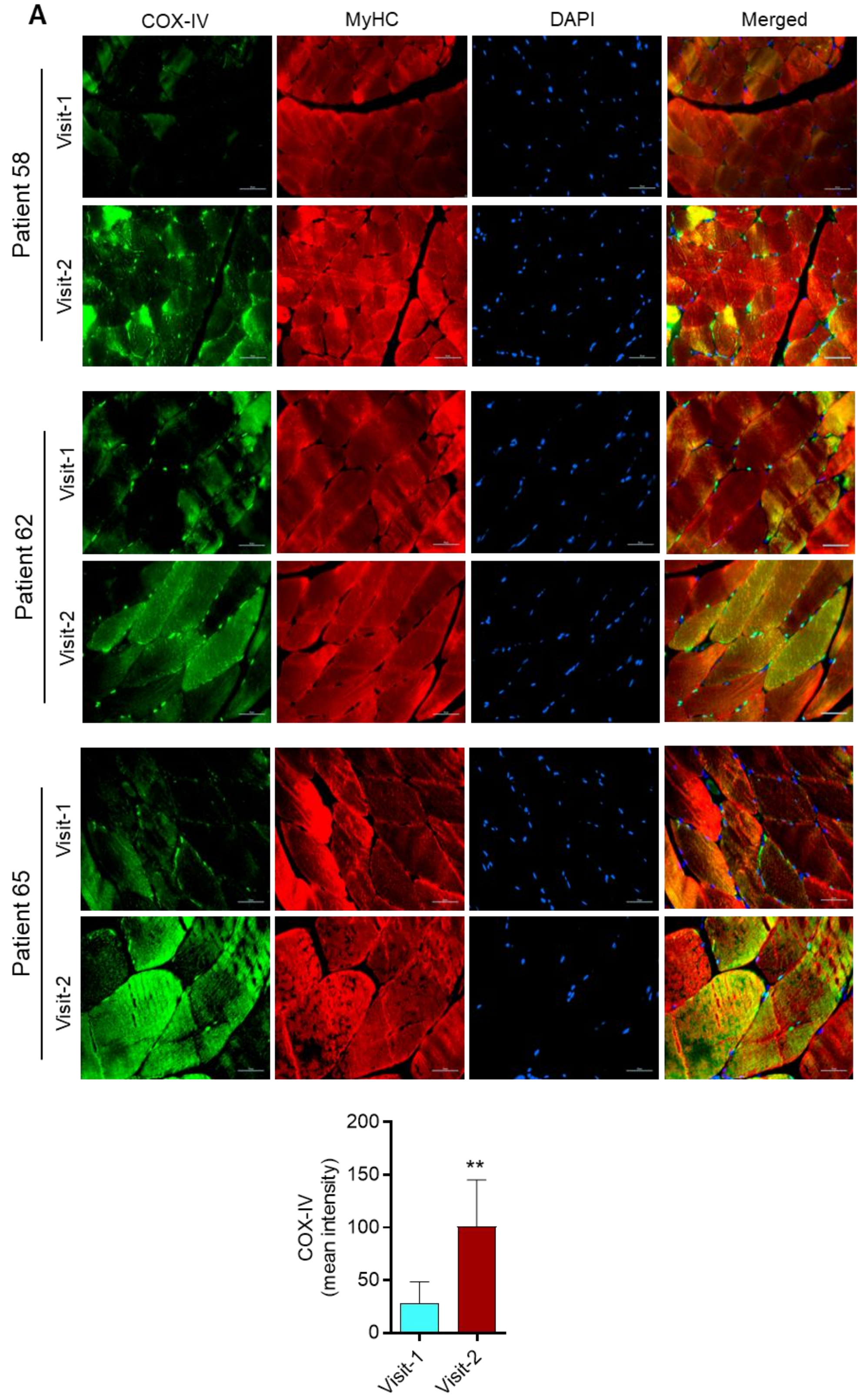
A
COX-IV
MyHC
DAPI
Merged
Patient 58
Visit-1
Visit-2
Patient 62
Visit-1
Visit-2
Patient 65
Visit-1
Visit-2
COX-IV
(mean intensity)
200
150
100
50
0
**
Visit-1
Visit-2

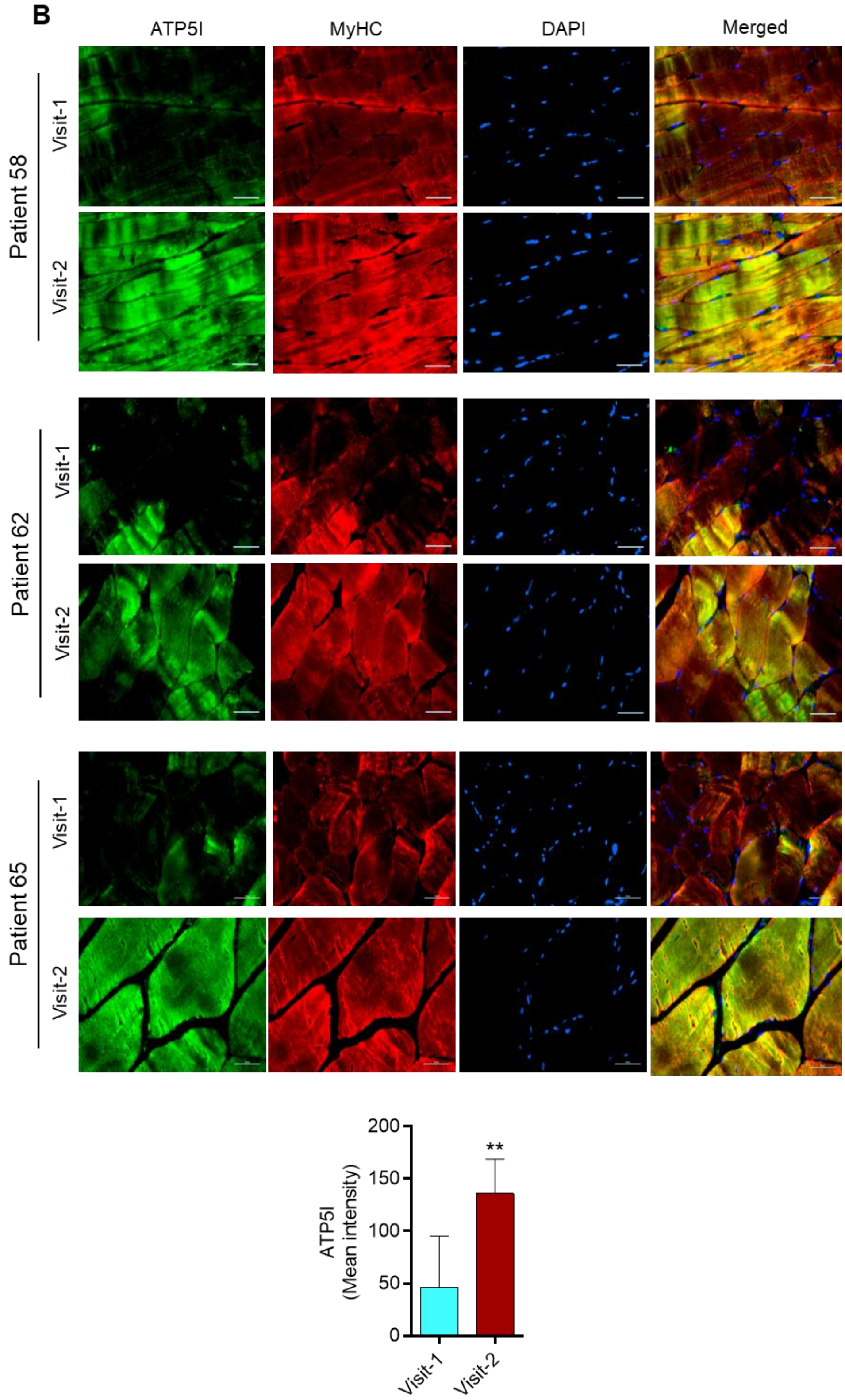
B
ATP5I
MyHC
DAPI
Merged
Patient 58
Visit-1
Visit-2
Patient 62
Visit-1
Visit-2
Patient 65
Visit-1
Visit-2
ATP5I (Mean intensity)
200
150
100
50
0
**
Visit-1
Visit-2

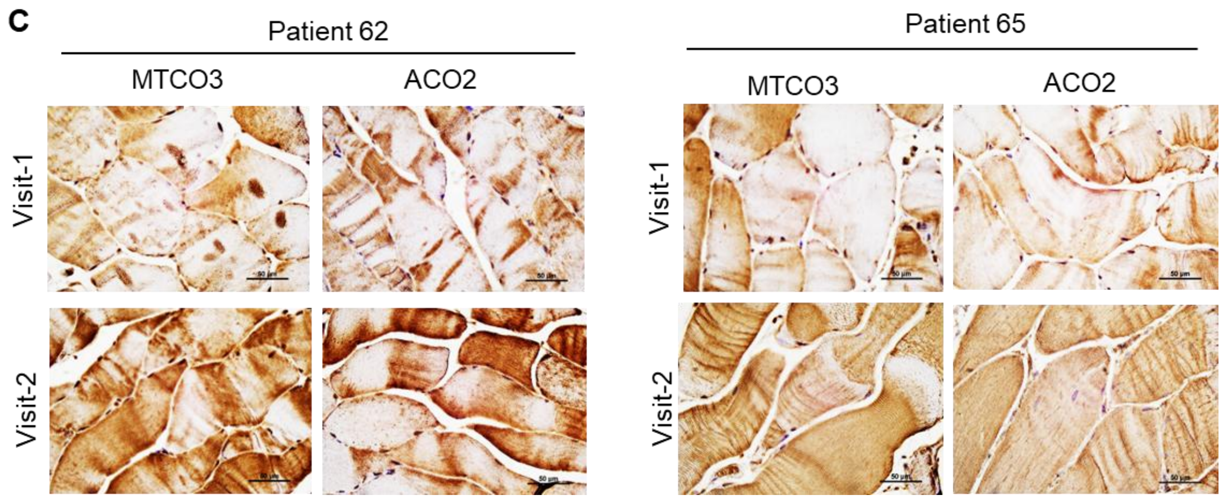
C
Patient 62
MTCO3
ACO2
Visit-1
Visit-2
Patient 65
MTCO3
ACO2
Visit-1
Visit-2

Figure S5

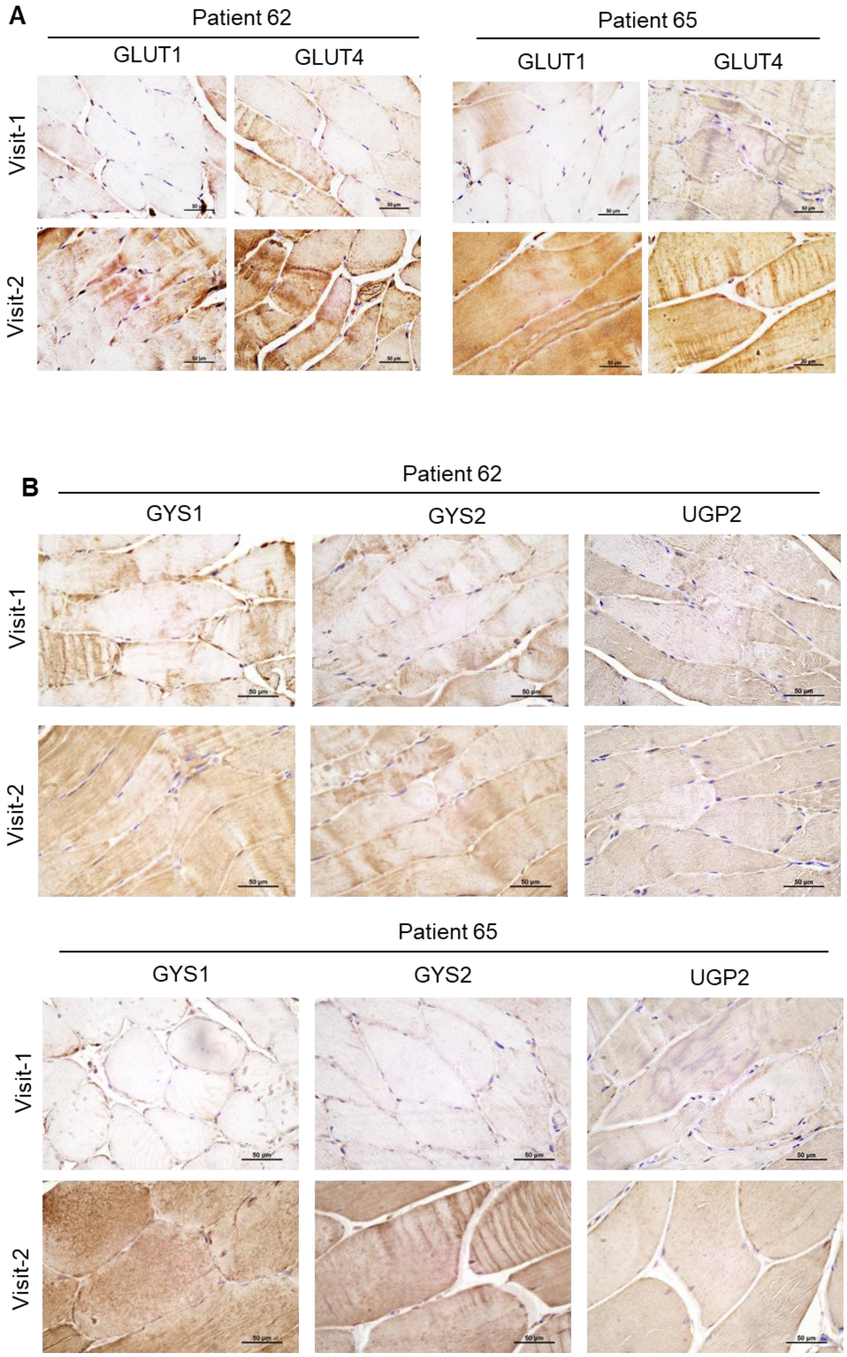
A
Patient 62
GLUT1
GLUT4
Patient 65
GLUT1
GLUT4
Visit-1
Visit-2
B
Patient 62
GYS1
GYS2
UGP2
Visit-1
Visit-2
Patient 65
GYS1
GYS2
UGP2
Visit-1
Visit-2

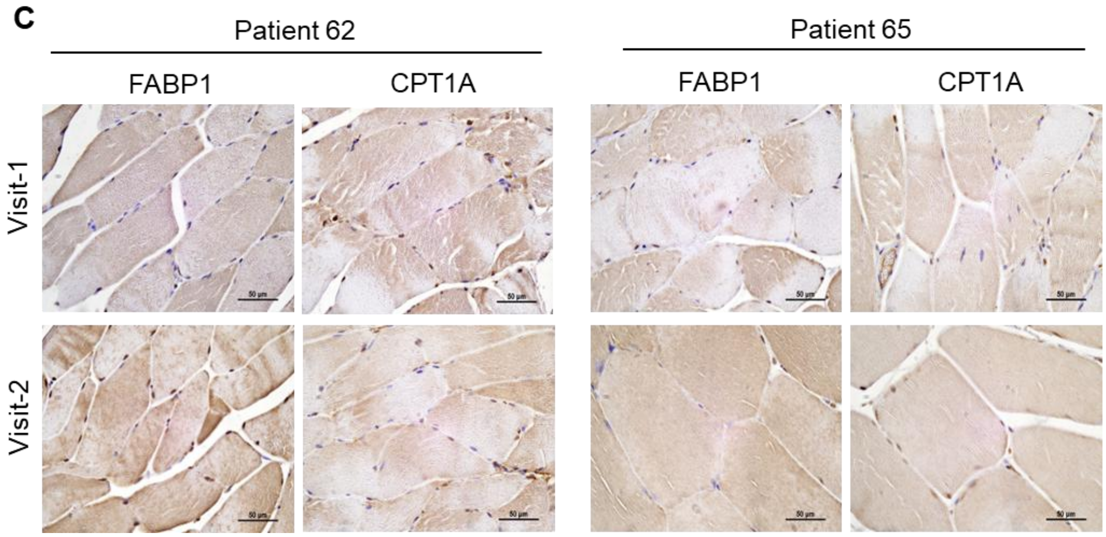
C
Patient 62
Patient 65
FABP1
CPT1A
FABP1
CPT1A
Visit-1
Visit-2
